# Re-Classifying Hypertension in the Venezuelan EVESCAM Database Using 2017 AHA/ACC Criteria: High Prevalence, Poor Control, and Urgent Call for Action

**DOI:** 10.5334/aogh.2346

**Published:** 2019-05-28

**Authors:** Juan González-Rivas, Jeffrey Mechanick, Maritza Duran, Eunice Ugel, María Marulanda, Ramfis Nieto-Martínez

**Affiliations:** 1 The Andes Clinic of Cardio-Metabolic Studies, Mérida, VE; 2 Icahn School of Medicine at Mount Sinai, New York, US; 3 Avila Clinic, VE; 4 School of Medicine, University Centro-Occidental “Lisandro Alvarado”, Barquisimeto, VE; 5 Endocrine Associates of Florida, Orlando, Florida, US; 6 South Florida Veterans Affairs Foundation for Research & Education and Geriatric Research, Education, and Clinical Center (GRECC), Miami VA Healthcare System, Miami, Florida, US

## Abstract

**Background:**

In 2017 the American Heart Association (AHA)/American College of Cardiology (ACC) changed the criteria to define hypertension (HTN).

**Objective:**

To re-analyze Venezuelan data to update HTN prevalence rates and estimate the number of adults with uncontrolled blood pressure (BP) using AHA/ACC criteria.

**Methods:**

The EVESCAM was a national population-based, cross-sectional, randomized cluster sampling study, which assessed 3,420 adults from July 2014 to January 2017, with a response rate of 77.3%. The mean of two BP measurements was obtained using a standard oscillometric device protocol. HTN was defined using both 2017 AHA/ACC guideline (BP ≥ 130/80 mmHg) and JNC7 (BP ≥ 140/90 mmHg) criteria.

**Findings:**

The crude prevalence of HTN using 2017 AHA/ACC guideline criteria was 60.4%, 13% higher than with the JNC7 criteria. The age-standardized prevalence was 55.4% in men and 49.0% in women (p < 0.001), 17.5% and 12.7% higher, respectively, compared with the JNC7 criteria. In subjects without self-reported HTN, the age-standardized prevalence of HTN was 43.4% in men and 32.3% in women, of whom, 22.9% and 19.2% were between 130–139/80–89 mmHg, respectively. In those with self-reported HTN, the prevalence of uncontrolled BP (≥130/80 mmHg) on antihypertensive medication was 66.8% in men and 65.8% in women. The total estimated number of subjects with HTN in Venezuela increased to 11 million, and only about 1.8 million are controlled.

**Conclusion:**

Using the new 2017 AHA/ACC guideline, the prevalence of HTN in Venezuela is approximately half of the adult population and associated with relatively poor BP control.

## Introduction

Hypertension (HTN) is the leading global burden modifiable risk factor, responsible for 211 million of DALYs (Disability-Adjusted Life Year) in 2015, followed by tobacco use and increased weight [1]. Blood pressure (BP) values over 115/75 mmHg expose a log-linear relationship with cardiovascular disease (CVD) [2]. Despite a linear relationship between BP and CVD, HTN was defined as SBP ≥ 140 mmHg or DBP ≥ 90 mmHg primarily based on observational studies, but also a cadre of randomized clinical trials [3]. However, the recent clinical practice guidelines presented by the American Heart Association (AHA) and the American College of Cardiology (ACC) redefines HTN diagnosis and subsequent BP target goals based on a 130/80 mmHg cutoff [4]. This conspicuous numerically based change is primarily based on the Systolic Blood Pressure Intervention Trial (SPRINT) [5], and supported by other relevant observational studies [2,6,7]. In the SPRINT study, 9361 subjects were randomly assigned to a target of <120 mmHg (intensive treatment) or <140 mm Hg (standard treatment). The study was stopped early after a median of follow-up of only 3.26 years. In the first year, the mean SBP was 121.4 mm Hg in the intensive group and 136.2 mmHg in the standard group. The primary composite outcome (myocardial infarction, stroke, heart failure, or death from CVD) was 25% lower (hazard ratio [HR] 0.75; 95% confidence interval [CI], 0.64–0.89), and all-cause mortality 27% lower (HR 0.73; 95% CI, 0.60–0.90), than in the intensive treatment group [5]. This benefits were higher in the >75 years age group, where the primary composite outcome was 34% lower (HR 0.66; 95% CI 0.51–0.85), and all-cause mortality 33% lower (HR 0.67; 95% CI, 0.49–0.91), without serious adverse events (HR, 0.99; 95% CI, 0.89–1.11) [8].

As consequence to this change in diagnostic and target criteria for HTN, re-analysis of the 2011–2014 National Health and Nutrition Examination Survey (NHANES) found that the crude prevalence of HTN in the U.S. increased from 31.9% (95% CI: 30.1–33.7) to 45.6% (95% CI: 43.6–47.6). However, only 1.9% of those newly diagnosed subjects with HTN would need antihypertensive medication because, in this new lower cutoff of HTN, the current management recommendation is mainly lifestyle change [9].

Venezuela is a nation undergoing severe, pervasive, and adverse economic, demographic, and cultural transformations that have overtly compromised public health and the ability for government and private parties to provide necessary health care services [10]. HTN plays a critical role in the pathogenesis of chronic disease, particularly CVD, which is the leading cause of death globally (including Venezuela) [11]. Not only will a better understanding of population-based hypertension parameters help guide the struggling Venezuelan health care system, but this process of re-classification of the HTN problem can be useful for other global scenarios, stable or unstable.

It stands to reason, that quantitating the impact of the 2017 AHA/ACC guidelines on HTN prevalence rates and control in Venezuela is critical to population-based strategies for health care and specifically CVD prevention. This study will re-analyze the Venezuelan data to update the HTN prevalence rates and estimate the number of adults with uncontrolled BP using the Venezuelan Study of Cardio-Metabolic Health (EVESCAM, for its acronym in Spanish) database.

## Methods

### Design

The study design, sampling, and implementation were described previously [12,13]. In brief, the EVESCAM was a population-based, observational, cross-sectional, and cluster sampling study, designed to evaluate cardiometabolic risk factors among subjects aged ≥20 years in Venezuela from July 2014 to January 2017. Unlike VEMSOLS, who assessed only three of the eight regions, the EVESCAM evaluated the entire country.

### Sampling and Recruitment

A multi-stage stratified sampling method was used to select a representative sample of the general population of Venezuela. 4454 women and men, aged 20 years and older, were recruited from randomly selected samples in the eight regions of Venezuela. Initially, 23 cities (1st stage) from the eight regions – one to four cities per region – were chosen. Each selected city was stratified by municipalities. Two municipalities (2nd stage) in each city, then two parishes (3rd stage) in each municipality, and finally two locations (4th stage) in each parish, were randomly selected. In the 5th stage, mappings and censuses of each location delimited the streets or blocks (primary sampling units) and selected the households to visit. Actual household visits were conducted in the 6^th^ stage. Inclusion criteria were all those subjects with 20 or older years of age living in the house selected for more than six months. Exclusion criteria were current pregnancy, inability to stand or communicate, or refusal to participate in the study by not signing the informed consent.

The sample size was calculated to detect a diabetes prevalence (the lowest prevalent condition reported in Venezuela) of 7.7% [14] with a standard deviation of 1.55% and a confidence level of 95%. The minimal estimated number of subjects to be evaluated was 2940. Considering a minimal expected response rate of 70%, the final sample size was 4200, representing the proportions of the country in terms of age, sex, race, social status, and proportion of rural and urban populations. 4454 subjects were recruited (86.3% urban and 13.7% areas), among which 3420 were evaluated for a net response rate of 77.3%.

The study protocol was designed in compliance with the Helsinki declaration and approved by the National Bioethics Committee (CENABI). Consent from all participants was obtained and filed. The present report is presented according to the Strengthening the Reporting of Observational Studies in Epidemiology (STROBE) [15]. A customized questionnaire was used to collect information on demographics, hypertension, and other cardiovascular risk factors. Physical measurements were obtained by trained and certified health personnel.

### Blood pressure measurements

BP was measured twice, with five minutes intervals, in the right arm, supported at heart level, in a sitting position, after five minutes of rest, with a validated oscillometric sphygmomanometer (Omron HEM-705C Pint® Omron Healthcare CO., Kyoto/Japan) [16]. The mean of the two measurements was used to define SBP and DBP.

### Definition of variables

Hypertension was defined using the 2017 AHA/ACC guideline [4] as SBP ≥130 mmHg, DBP ≥80 mmHg, or self-report of hypertension or antihypertensive medication use. Subjects without a prior diagnosis of HTN were classified as “normal” if SBP was <120 mmHg and DBP was <80 mmHg; “elevated” if SBP was from 120 to 129 mmHg and DBP was <80 mmHg; “HTN stage 1” if SBP was from 130 to139 mmHg or DBP was from 80 mmHg to 89 mmHg; or “HTN stage 2” if SBP or DBP was ≥140/90 mmHg. Subjects with a prior diagnosis of HTN with SBP ≥ 130 mmHg or DBP ≥ 80 mmHg were classified as “uncontrolled” and those with SBP <130 mmHg and DBP <80 mmHg as “controlled”. Diagnostic classifications using the Seventh Report of the Joint National Committee on Prevention, Detection, Evaluation and Treatment of High Blood Pressure (JNC7), as SBP ≥ 140 mmHg, DBP ≥ 90 mmHg, or self-report of hypertension or antihypertensive medication use, were also provided for each subject.

### Data analysis

All calculations were performed using SPSS 20 software (IBM Corp. Released 2011. Armonk, NY, USA). Continuous variables were initially tested for normality (Q-Q plots). Age, SBP, and DBP were presented as a mean ± standard error of the mean (SEM), and their differences were assessed by Student t-test. Prevalence was presented as percent and 95% confidence intervals (95% CI). The population was divided into seven age groups (20–29; 30–39; 40–49; 50–59; 60–69; 70–79; 80+). Lost values were less than 1% and no adjustment was required. To enable comparison with other global populations, age and sex direct standardization using the World Health Organization world population was used [17]. Prevalence differences were compared using the Chi-square test. A p-value <0.05 was considered statistically significant. To estimate the number of subjects with HTN, the population size reported by the Venezuelan National Institute of Statistics (www.ine.gov.ve; accessed on January 21, 2018) was used: 31,431,164 inhabitants in 2017, of whom 65.3% were 20 years or older, and 50% were female.

## Results

### Population studied

Two-thirds of the study subjects were female. Men had higher age and BP than women (Table 1).

**Table 1:** Subjects characteristics by gender.

	Men	Women	Total

n (%)	1064 (31.1)	2356 (68.9)	3420 (100.0)
Age (years)^†^	51.6 ± 0.33	49.6 ± 0.33	50.2 ± 0.27
Systolic BP (mmHg)^†^	134.9 ± 0.69	129.4 ± 0.49	131.2 ± 0.40
Diastolic BP (mmHg)^†^	77.5 ± 0.38	76.4 ± 0.23	76.7 ± 0.20

Data are presented as mean ± SEM. ^†^ Differences between means using Student t-test p < 0.001. Abbreviations: BP – Blood pressure.

### Prevalence of hypertension

**Figure 1:**
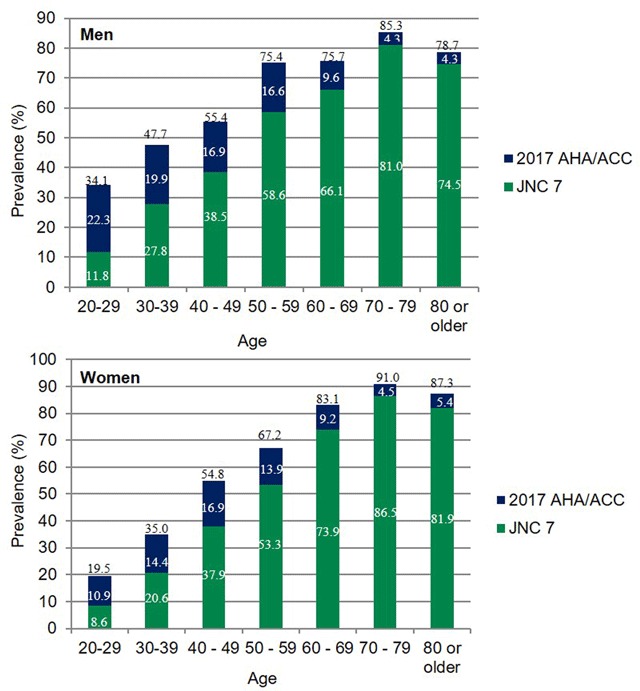
Prevalence of hypertension by age according to the 2017 AHA/ACC and JNC7 definitions in men and women.

The crude prevalence of HTN using the 2017 AHA/ACC guideline criteria was 60.4%, 13% higher than with the JNC 7 criteria (Table 2). The age-standardized prevalence was 55.4% in men and 49.0% in women (p < 0.001), 17.5% and 12.7% higher, respectively, compared with the JNC 7 criteria. The prevalence of hypertension increased with age until the 8^th^ decade in both genders (p < 0.001). The 2017 AHA/ACC definition increased the detection of subjects with HTN, especially in younger people (Figure 1). The proportion of newly detected subjects with HTN was 65.4%, 42.7%, 30.5%, 22.1%, 12.7%, and 5% in men aged 20–29, 30–39, 40–49, 50–59, 60–69 and ≥70 year old groups, respectively. There were similar proportions in women. HTN presented in 7 of out 10 men after the fifth decade of life and in 8 out of 10 women after the seventh decade of life.

**Table 2:** Comparative Crude and Age-Standardize Prevalence by Gender According to the 2017 AHA/ACC and JNC 7 Guidelines.

Guideline	2017 AHA/ACC		JNC 7	

Definition used	SBP/DBP ≥ 130/80 mmHg or self-reported hypertension		SBP/DBP ≥ 140/90 mmHg or self-reported hypertension	

Overall, crude	60.4 (58.7–62.0)		47.4 (45.7–49.0)	
	**Men**	**Women**	**Men**	**Women**

Overall, age-sex standardize	55.4 (52.4–58.3)	49.0 (46.9–51.0)	37.9 (33.8–42.1)	36.3 (33.5–39.2)

Age group				
20–29	34.1 (25.8–42.3)	19.5 (15.0–23.9)	11.8 (6.1–14.4)	8.6 (5.4–11.7)
30–39	47.7 (39.7–55.6)	35.0 (30.2–39.7)	27.8 (20.6–34.9)	20.6 (16.5–24.6)
40–49	55.4 (48.2–62.3)	54.8 (50.2–59.2)	38.5 (31.6–45.3)	37.9 (33.4–42.3)
50–59	75.2 (69.3–81.0)	67.2 (63.2–71.1)	58.6 (51.9–65.2)	53.3 (49.1–57.4)
60–69	75.7 (70.0–81.3)	83.1 (79.5–86.6)	66.1 (59.8–72.3)	73.9 (69.7–78.1)
70–79	85.3 (78.8–91.7)	91.0 (86.8–95.2)	81.0 (73.8–88.1)	86.5 (81.4–91.5)
80 or older	78.7 (66.9–90.4)	87.3 (79.6–94.9)	74.5 (62.0–86.9)	81.9 (73.0–90.7)

Data are presented as a percent and 95 CI. Differences between genders were calculated using Chi-Square. Abbreviations: ACC – American College of Cardiology; AHA – American Heart Association; DBP – Diastolic Blood Pressure; SBP – Systolic Blood Pressure.

### New staging of hypertension prevalence and uncontrolled hypertension

**Figure 2:**
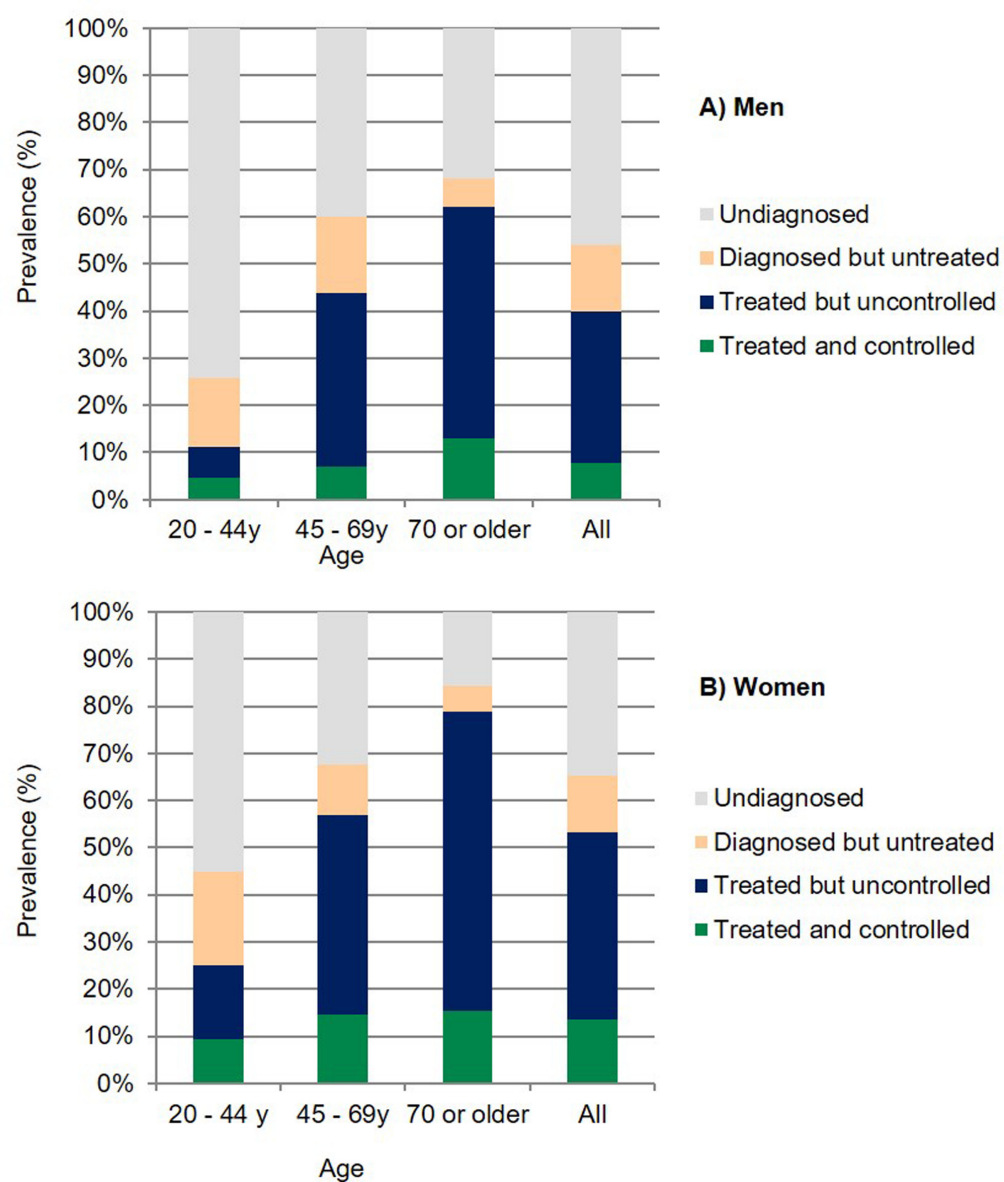
Prevalence of undiagnosed, treated, and controlled subjects with hypertension by age and gender.

In subjects without self-report of HTN, the age-standardized prevalence of HTN was 43.4% in men and 32.3% in women, of whom, 22.9% and 19.2% were stage 1, respectively (Table 3). The subjects in stage 1 represent those recently included in the diagnosis of HTN, around 1.7 million men and 1.3 million women. In this stage, more than 90% require lifestyle intervention only. Pharmacological antihypertensive treatment is recommended in those subjects with diabetes or with a 10-year cardiovascular risk ≥10%, representing 164,000 men and 78,000 women in the present study. The estimated number of adults that required initiation of antihypertensive medication in 2017 was 2.7 million (Table 3). In those with self-reported HTN, the prevalence of uncontrolled BP (≥130/80 mmHg) on antihypertensive medication was 66.8% in men and 65.8% in women, representing an estimated 3.6 million of those with uncontrolled HTN. The total estimated number of subjects with HTN in Venezuela has increased to 11 million, and only about 1.8 million are controlled, representing 16.6% of subjects with HTN. The prevalence of subjects with HTN, undiagnosed, treated, and controlled by age and gender is summarized in Figure 2.

**Table 3:** Age-standardized Prevalence and Estimated Number of Adults with Hypertension and Uncontrolled Hypertension in Venezuela According to the 2017 AHA/ACC Guideline by Gender.

Subjects without self-report of hypertension (n = 2147)		

	Men (n = 694)	Women (n = 1452)

**Classification of blood pressure**		
**Normal** (%) (<120/80 mmHg)	33.3	53.9
Estimated number	2,576,673	3,910,676
**Elevated** (%) (120–129/<80 mmHg)	23.4	13.8
Estimated number	1,810,635	1,001,249
**Hypertension Stage 1** (%) (130–139/80–89 mmHg)	22.9	19.2
Estimated number	1,771,946	1,393,042
Recommended only lifestyle treatment (%)	90.7	94.4
Estimated number	1,607,155	1,315,032
Recommended pharmacological treatment (%)	9.3	5,6
Estimated number	164,791	78,010
**Hypertension Stage 2** (%) (≥140/90 mmHg)	20.4	13.1
Estimated number	1,578,502	950,461
Total number of subjects that require initiate antihypertensive medication (Stage 2 + Stage 1 with 10y CV risk ≥10% or DM)		2,771,764
**Subjects with self-report of hypertension (n = 1272)**		

	**Men (n = 369)**	**Women (n = 903)**

Age-standardized prevalence of self-reported hypertension (%)	24.6	29.3
Estimated number	2,524,520	3,006,847
Prevalence of treated and uncontrolled hypertensive adults (≥130/80 mmHg)	66.8	65.8
Estimated number	1,686,379	1,978,505
Total number of hypertensive adults (Self-reported + Stage 1 + Stage 2)	5,874,968	5,350,350
Total (both sex) number of hypertensive adults		11,225,318

Abbreviations: AHA – American Heart Association; ACC – American College of Cardiology; CV – Cardiovascular risk; DM – Diabetes mellitus.

## Discussion

Using the 2017 AHA/ACC guideline [4], the prevalence of HTN in Venezuela is extremely high (60.2%). This reflects an enormous public health problem, aggravated by a fractured and dysfunctional health care system, and therefore posing a heavy challenge to health care professionals, policy makers, and other stakeholders now charged with fashioning a realistic solution [18]. The Assessment Capacities Project (ACAP) declared Venezuela in humanitarian crisis and expected to continue in the following years [19]. The main drivers to this crisis are the economic situation, the continued erosion of democratic institutions, and insecurity [19]. By the end of 2018, the inflation rate was 1.37 million percent and is expected to be roughly 10 million percent in 2019 [20]. This astronomical economic burden profoundly impairs access and delivery of basic health care services, with medicine shortages exceeding 90%, and other costs far outstripping any reasonable expectation of resources.

According to this report, more than half of Venezuelan adults have HTN, representing around 11 million people, of which nearly 3 million needs to start antihypertensive medication, though only 1.8 million were actually treated and controlled (< 130/80 mmHg). This new cut-off value using the 2017 AHA/ACC criteria to diagnose HTN increases the ability to detect subjects at risk that can also benefit from intervention, especially those < 50 years old, in whom the prevalence rate is 30–60%.

Analyzing the 2011–2014 NHANES database (n = 9,623), the prevalence of HTN in the U.S. increased from 31.9% (95% CI 30.1%–33.7%) using the JNC-7 to 45.6% (95% CI 43.6%–47.6%) using the 2017 AHA/ACC guideline. Compared with Venezuela, the prevalence of HTN in the U.S. was 6.8% and 6.2% lower in men and women, respectively. Similarly, the proportion of subjects with stage 1 HTN that require pharmacological treatment in the U.S. is substantially lower than Venezuelan adults (1.9% vs. 6.0%; stage 1 with high CVD risk). Among those taking antihypertensive medication in the U.S., 53.4% were above the treatment goal according to the 2017 AHA/ACC (<130/80 mmHg), lower than that observed in Venezuela ≈66%. In summary, compared with the U.S., which is the only published data for the moment with the 2017 AHA/ACC guideline, Venezuela has a larger proportion of adults with HTN, with more requiring pharmacological treatment at stage 1, and less being controlled to target.

This large number of adults with HTN is problematic for the highly compromised Venezuelan public health system. Cardiovascular diseases are the leading cause of death in Venezuela, and if these 11 million adults with HTN are not properly managed, CVD prevalence rates, related disabilities, and costs will balloon. It is estimated that almost 3 million adult Venezuelans will need to start anti-hypertension medical therapy, while another 3.6 million will need to adjust their medicines.

Venezuelans are exposed to a stressful environment as a consequence to chronic social, political, and economic turmoil [21]. Hypothalamic stress responses activate the sympatho-adrenal and pituitary-adrenal axes. Over time, resultant counter-regulatory factors increase cardiac output and peripheral vascular resistance, elevating BP and creating a hypertensive physiological state [22]. However, this complex stress response varies from person to person. A meta-analysis, including 34,556 subjects in six cohort studies, reported that those who had stronger responses to stressors had a 21% higher risk of HTN than those with weaker responses (Odds Ratio [OR] 1.21; 95% CI, 1.14–1.28) [23]. In another study, after three years of follow up of 479 healthy adults who received laboratory-induced mental stress, HTN incidence was 59% higher (OR 1.59; 95% CI, 1.17–2.17) in those with higher salivary cortisol in response to mental stressors [24]. Also, low financial status, which affects most Venezuelans, is also a well-recognized factor related to HTN risk via chronic stress [25].

Some limitations of this study require discussion. The diagnosis of HTN was based only on two measures, and the current guideline recommends several measures. However, a rigorous measurement process was carried out across the study, and a validated automatic device was used to reduce bias. In the study, more women participated, which is typically observed in the surveys in this region where women tend to be more worried by their health than men; however, all the analysis were adjusted by gender. The use of drugs or external drivers that can modify the prevalence of HTN was not assessed.

In conclusion, using the new 2017 AHA/ACC guideline, the prevalence of HTN in Venezuela is approximately half of the adult population, and is associated with a low proportion of BP control. It is urgent that challenges confronted, strategies formulated, and public health policies implemented to reduce HTN incidence and prevalence in Venezuela.
